# DNA fragmentation in spermatozoa: a historical review

**DOI:** 10.1111/andr.12381

**Published:** 2017-07-17

**Authors:** A. S. Rex, J. Aagaard, J. Fedder

**Affiliations:** ^1^ Aagaard Gynaecological Clinic Aarhus Denmark; ^2^ Centre of Andrology & Fertility Clinic Odense University Hospital Odense Denmark; ^3^ Department of Clinical Research University of Southern Denmark Odense Denmark

**Keywords:** artificial reproductive treatments, chromatin structure, DNA fragmentation, intra uterine insemination, male infertility, spermatozoa

## Abstract

Sperm DNA Fragmentation has been extensively studied for more than a decade. In the 1940s the uniqueness of the spermatozoa protein complex which stabilizes the DNA was discovered. In the fifties and sixties, the association between unstable chromatin structure and subfertility was investigated. In the seventies, the impact of induced DNA damage was investigated. In the 1980s the concept of sperm DNA fragmentation as related to infertility was introduced as well as the first DNA fragmentation test: the Sperm Chromatin Structure Assay (SCSA). The terminal deoxynucleotidyl transferase nick end labelling (TUNEL) test followed by others was introduced in the nineties. The association between DNA fragmentation in spermatozoa and pregnancy loss has been extensively investigated spurring the need for a therapeutic tool for these patients. This gave rise to an increased interest in the aetiology of DNA damage. The present decade continues within this research area. Some of the more novel methods recently submerging are sorting of cells with increased DNA fragmentation and hyaluronic acid (HA) binding techniques. The clinical value of these tests remains to be elucidated. In spite of half a century of research within the area, this analysis is not routinely implemented into the fertility clinics. The underlying causes are multiple. The abundance of methods has impeded the need for a clinical significant threshold. One of the most promising methods was commercialized in 2005 and has been reserved for larger licensed laboratories. Myriads of reviews and meta‐analyses on studies using different assays for analysis of DNA fragmentation, different clinical Artificial Reproductive Treatments (ART), different definitions of successful ART outcome and small patient cohorts have been published. Although the area of DNA fragmentation in spermatozoa is highly relevant in the fertility clinics, the need for further studies focusing on standardization of the methods and clinical implementation persists.

## Introduction

Paternal contribution to the fertilization and to the development of healthy offspring is of vital importance. There have been reports of an increased risk of schizophrenia or autism in offspring from fathers with increasing age (Sipos *et al*., 2004; Reichenberg *et al*., 2006) and an increased risk of cancer in offspring from fathers with increased level of sperm DNA fragmentation because of smoking (Ji *et al*., 1997). Furthermore, some spontaneous dominant genetic diseases, epilepsy and some birth defects are linked to paternal contribution (reviewed in Aitken *et al*., 2009). In a number of studies, an association between increased DNA fragmentation in the spermatozoa and subfertility has been reported. Comparing studies of fertile and infertile males have shown that the amount of DNA fragmentation is significantly higher in the infertile group (Evenson *et al*., 1999; Spano *et al*., 2000; Saleh *et al*., 2003; Alkhayal *et al*., 2013; Oleszczuk *et al*., 2013). An abnormal chromatin packing is more recurrent in men with normozoospermia undergoing ART treatment than in fertile men (Alkhayal *et al*., 2013). If the man has increased DNA fragmentation in the spermatozoa, a prolonged Time To Pregnancy (TTP) (Evenson *et al*., 1999), an increased risk of a missed abortion (Virro *et al*., 2004; Lin *et al*., 2008; Zini *et al*., 2008; Kennedy *et al*., 2011; Dar *et al*., 2013) and a significantly reduced success rate in in vivo fertilization of the partner have been observed (Spano *et al*., 2000; Bungum *et al*., 2004, 2007; Giwercman *et al*., 2010; Zini, 2011). When seeking fertility treatment, it seems that sperm DNA fragmentation is of vital importance when planning the course of treatment. A study included 131 couples seeking fertility treatment by intrauterine inseminations (IUI). Twenty‐three of the male patients had an increased amount of SCSA defined DNA fragmentation followed by a pregnancy rate of 4% in their partner (Bungum *et al*., 2004). A later study including 387 cycles showed that the pregnancy rate dropped to 3% if the level of DNA fragmentation exceeded 30% (Bungum *et al*., 2007). In a smaller Danish study including 48 couples, no pregnancies were observed in couples, where the male DNA fragmentation exceeded 27% (Boe‐Hansen *et al*., 2006). DNA fragmentation may affect the fertilization rate after in vitro fertilization (IVF). No clear association between increased amount of DNA fragmentation and intracytoplasmic sperm injection (ICSI) has been established. However, DNA fragmentation may affect the clinical pregnancy rate (Oleszczuk *et al*., 2016) (Bungum *et al*., 2007; Dar *et al*., 2013).

It thus seems that an increase in DNA fragmentation primarily affects in vivo fertility, either by reducing natural conception or by a significant reduction in successful intrauterine inseminations. It is estimated that up to 20% of males with semen parameters otherwise suitable for IUI treatment present with a DFI ˃30%. On this basis, the authors behind this study recommend that IVF or ICSI being the first choice of treatment if the amount of DNA fragmentation exceeds 30% (Giwercman *et al*., 2010; Bungum *et al*., 2011). However, in the study from Oleszczuk *et al*. (2016) it was found that the fertilization rates might also be decreased after IVF when DFI by SCSA exceeds 30%. For high degree of DFI, it thus might be relevant to proceed the treatment using ICSI (Oleszczuk *et al*., 2016).

Together, these studies provide important insight into the significance of sperm DNA fragmentation when treating couples for infertility.

With this in mind ‐ why is sperm DNA fragmentation testing not a standard diagnostic tool in the treatment of the male fertility patient?

The journey regarding DNA fragmentation in spermatozoa has been long and began more than half a century ago.

## Historical Overview

### Forties, fifties and sixties

In 1946, Pollister and Mirsky discovered that a large part of the protein complexes surrounding the DNA in trout sperm was not composed of histones but of protamines (Pollister & Mirsky, 1946). Later Alfert found that the protamines replace the histones after meiosis in the maturation of the salmon spermatozoa (Alfert, 1956). Today it is estimated that only 5–15% of the chromatin in the human spermatozoa consist of histones and the major part consists of protamines (Castillo *et al*., 2015). Alongside the discovery of the double helix in 1953, Leuchtenberger *et al*. (1953) discovered that the amount of DNA from infertile males had a significantly larger variation compared with fertile males. Already at this time, it was discovered that the quality of a sperm sample was more than a question of number and motility of the spermatozoa (Leuchtenberger *et al*., 1953).

### The seventies

During the seventies, an increasing interest in a possible association between exposure of DNA damaging agents and a possible reduction in fertility emerged. In 1970, Ringertz *et al*. used an assay where bull spermatozoa were heated and the subsequent denaturation of the DNA was detected with acridine orange followed by microfluoriemetry. They realized that the spermatozoa possessed an increased stability during the spermiogenesis (Ringertz *et al*., 1970). A decrease in epididymal sperm count and weight of the testis was observed in mice after exposure to irradiation. Subsequently, an increased pre‐implantation loss was observed in the female mice (Searle & Beechey, 1974).

### The eighties

In the eighties, the technology for molecular biology advanced. Evenson *et al*. developed a flow cytometric assay for detection of DNA fragmentation in spermatozoa (Evenson *et al*., 1980). They called the assay Sperm Chromatin Structure Assay (SCSA). The assay is based on the detection of DNA fragmentation by flow cytometry. The sperm DNA is denaturized by acid at sites of DNA strand breaks and subsequent stained with the fluorescent cationic dye Acridine Orange (AO). In this assay, AO attaches to the DNA in the ratio of approximately two AO molecules per phosphate group (Evenson & Jost, 1994). When the laser from the flow cytometer illuminates the cells, AO fluoresces with a green emission when bound to double stranded (db) DNA and a red emission when bound to singe stranded (ss) DNA. Furthermore, the flow cytometer measures forward scatter and side scatter of the sample and this can help exclude debris from the sample. Usually a total of 5000–10,000 cells are analysed. DNA Fragmentation Index (DFI) is described as the percent wise ratio of red florescence to green + red fluorescence (Larson *et al*., 2000; Evenson *et al*., 2002; Larson‐Cook *et al*., 2003). SCSA also measures High DNA Stainability (HDS), which is believed to be an expression of immature spermatozoa containing excess histones or other abnormal proteins (Evenson *et al*., 2002; Bungum *et al*., 2004).

### The nineties

The field of single cell electrophoresis was developed in the eighties and optimized during the nineties making it possible to detect DNA fragmentation by the comet assay. The comet assay was a novel diagnostic tool for DNA fragmentation and was used to emphasize that spermatozoa from infertile men were more susceptible to induced damage than spermatozoa from fertile men. In the comet assay, 200–300 cells are covered with agarose gel and subsequently lysed. If the DNA is embedded with breaks, the supercoiling of the DNA is released allowing the DNA to migrate towards the anode. This migration leaves a comet‐like tail and the fluorescent intensity of the tail relates to the number of DNA breaks (Hughes *et al*., 1996; Aravindan *et al*., 1997).

In the nineties, TUNEL used to detect DNA fragmentation in human spermatozoa was developed. In this assay a terminal deoxynucleotidyl transferase labels the DNA strand breaks with fluorescent dUTP nucleotides. The assay can be performed using either flow cytometry or microscopy. Thus, both the neutral and alkaline comet assay and the TUNEL assay are considered ‘direct’ assays as they measure actual DNA strand breaks, whereas some of the other assays developed measure the DNA susceptibility to denature at sites of ss or ds DNA breaks or a differentiated binding of a dye to ds‐ or ssDNA (Gorczyca *et al*., 1993; Zini & Sigman, 2009; Henkel *et al*., 2010).

### The zeroes

In the zeroes, other methods for determination of DNA fragmentation appeared. The DNA Breakage Detection‐Florescence in situ Hybridization (DBD‐FISH) was developed for human spermatozoa. In this assay, the spermatozoa are fixed in an agarose matrix and the DNA is transformed into ssDNA by an alkaline unwinding solution. After the proteins are removed, the DNA is made accessible to hybridization with relevant probes that highlight the area for analysis. If the DNA strand contains increased amount of DNA breaks more probes will hybridize resulting in an increased fluorescence. This technique can be used for detecting DNA damage within specific sequence areas (Fernandez *et al*., 2000). The Sperm Chromatin Dispersion (SCD) test was also developed in this decade and was used to detect spermatozoa with increased amount of DNA fragmentation. In this assay, the spermatozoa are embedded in an agarose matrix and exposed to a lysing solution. The relaxed DNA loops prevent dispersion into the surrounding area. With specific DNA fluorochromes and a fluorescence microscope, the dispersed DNA is seen as a halo surrounding the nuclei. If the DNA is fragmented, little dispersion is seen resulting in a small halo. This makes it possible to detects spermatozoa with increased amount of DNA fragmentation (Fernandez *et al*., 2003). Two years later, an advanced SCD test was developed as a kit, Halosperm^®^ (Fernandez *et al*., 2005).

In 2006, Li *et al*. developed the ɣH2AX assay to determine double strand breaks in human spermatozoa. The assay takes advantage of the fact that some protein‐kinases induce phosphorylation of Ser139 on the histone H2AX. Phosphospecific antibodies are able to recognize the phosphorylated serine residue. These are subsequent quantified by a flow cytometer. Although most histones are replaced by protamines in human spermatozoa during the spermatogenesis, a small fraction remains in the nucleosome (around 15%). This fraction also contains the H2AX histone. In a recent study in 2015, Garolla *et al*. investigated the predictive value of the method. The pregnancy rate after ICSI was investigated and the method was compared with TUNEL. In this study, it was seen that the ɣH2AX percentage was higher in the males from non‐pregnant couples. This study also showed that the ɣH2AX has a better predictive value than TUNEL. In the present form, the method seems to be rather time consuming, as samples need more than 3 h of preparation before the flow cytometric analysis can be performed. This compared to the strict protocol from SCSA where samples can be prepared within few minutes (Li *et al*., 2006; Garolla *et al*., 2015; Evenson, 2016).

### The tenths

From the late zeroes and into the tenths the focus concerning DNA fragmentation shifted from development of methods to the aetiology of sperm DNA fragmentation. Furthermore, it became more evident that increased DNA fragmentation could be a valuable tool when deciding which type of fertility treatment the couple should be offered. Several studies added to the viewpoint that fertility treatment with IUI had very low chance of resulting in pregnancy if the SCSA‐DFI in the spermatozoa was increased. However, the implantation rate after ICSI is not affected by increased amount of DNA fragmentation in the spermatozoa, it has been seen that the risk of early pregnancy loss is increased in these couples (Carrell *et al*., 2003; Borini *et al*., 2006; Gil‐Villa *et al*., 2009; Brahem *et al*., 2011; Absalan *et al*., 2012; Oleszczuk *et al*., 2016).

In 2005, Greco *et al*. showed that ICSI with testicular sperm resulted in a significantly higher clinical pregnancy rate compared with ICSI where ejaculated sperm was used. This provided one of the first treatment options for male fertility patients with increased sperm DNA fragmentation. This study also gave insight to the aetiology of DNA damage as at least a part of the DNA damage seemed to appear after the spermatozoa have left the testis (Greco *et al*., 2005). Recently, both Esteves *et al*. and Pabuccu *et al*. achieved similar results. Other methods such as intracytoplasmic morphologically selected sperm injection (IMSI) and motile sperm organelle morphology examination (MSOME) have been used in order to circumvent the negative effects of increased DNA fragmentation. These methods are based on a real time examination of the spermatozoa under an increased magnification (up to × 13.000) which makes it possible to choose spermatozoa with better chromatin status and lower aneuploidy rate. If the spermatozoa showed a lack of vacuoles, the results improved further (Garolla *et al*., 2008, 2014; Gosálvez *et al*., 2013). In a recent study, Bradley *et al*. compared fertilization rates after physiological ICSI (PICS), IMSI and extraction of testicular sperm, respectively. They found that the use of testicular sperm significantly increased the fertilization rate, pregnancy rate and live birth rate after ICSI for patients with increased DNA fragmentation (Bradley *et al*., 2016).

### DNA fragmentation and infertility

A substantial amount of literature that strengthened the theory of a connection between increased DNA fragmentation and infertility had been published, and in 2015 Zini concluded that testing for DNA fragmentation should be a part of the routine male infertility diagnosing (Esteves *et al*., 2015; Zini, 2015; Pabuccu *et al*., 2016).

As the research in the area expanded, several studies show that the origin of DNA fragmentation can be very diverse. Link has been seen between increased DNA fragmentation and inadvertent effects during the spermiogenesis, increased amount of oxidative stress, sperm collection methods, storage temperature, varicocoele, bacterial infections, age, temperature of the testes and reaction to medicine (reviewed in Gonzalez‐Marin *et al*., 2012). It is thus possible that the damage to the DNA happens in multiple steps. This has probably contributed to the blurred picture of DNA fragmentation. One theory is that the DNA is subjected to damaging events during the spermatogenesis. This could include nicks in the backbone of the DNA or poor packaging of the chromatin during the replacement of histones. Subsequently, the already weakened DNA is more susceptible to external stressors such as medication, temperature and Reactive Oxygen Species (ROS) (McPherson & Longo, 1993; Pradeepa & Rao, 2007).

### Antioxidants

The role of antioxidants has been studied extensively in several areas in the last decade. Regarding spermatozoa, ROS are believed to play a part in the presence of DNA fragmentation. ROS play a positive role in several crucial functions such as proliferation and differentiation of cells. However, a pathogenic effect can occur when the balance between ROS and antioxidants are disturbed. This can result in an excess of ROS, for example in the reproductive tract or in the seminal plasm. It has been shown in several studies that antioxidants can have a positive impact on some of the primary seminal parameters (Zini *et al*., 2009; Abad *et al*., 2013; Agarwal *et al*., 2014). The distribution of dietary supplements to males with increased DFI have previously shown a significant reduction in DFI and an increase in the clinical pregnancy rate (Wright *et al*., 2014). However, the overall effect of antioxidants remains controversial. This is mainly because of non‐standardized assays for determination of ROS or antioxidant capacity, diversity in methods for determination of DNA fragmentation, lack of distinction between direct and indirect antioxidants and inadequate data on fertilization and pregnancy rates (Chen *et al*., 2013).

### Novel methods

In the present decade, the magnetic activated cell sorting (MACS) technique was enhanced. This method was used for the first time in connection to fertility treatment in the zeroes (Paasch *et al*., 2007). In this assay, magnetic particles conjugated to proteins or antibodies target the cells of interest. This could be apoptotic surface markers like externalized phospholipid phosphatidylserine (PS). PS has a high affinity for annexin V, which cannot cross the membrane. Any conjugation between the two will therefore happen on spermatozoa with externalized PS, which is seen in apoptotic cells. The magnetic particles, and thereby the apoptotic cells, can subsequently be removed by a magnetic cell separation column (Said *et al*., 2008).

A novel method where spermatozoa with increased amount of DNA fragmentation are separated by fluorescence‐activated cell sorting has recently been presented. The spermatozoa are stained using a YO‐PRO staining technique. The researchers behind the study showed that it is possible to separate the dead spermatozoa and the spermatozoa with increased amount of DNA fragmentation from the normal spermatozoa. This makes it possible to optimize the sperm sample before fertility treatment like ICSI is initiated (Ribeiro *et al*., 2013). However, recent research has shown that methods where ICSI is optimized remains controversial (Tavalaee *et al*., 2012; Troya & Zorrilla, 2015). Another novel method being investigated in the present decade is the possibility of detecting damage in spermatozoa by oligopeptides. A synthetic oligopeptide binds to the damaged DNA. The non‐binding end of the oligopeptide consists of a rhodamine B dye that can be detected with fluorescence microscopy. There was seen a correlation of the amount of DNA damage detected with this method and the more classical methods such as SCD, comet and TUNEL (Enciso *et al*., 2012). Another novel method that has been studied in the present decade is the HA binding technique. HA surrounds the oocyte only allowing spermatozoa with sufficient expression of specific receptors to fertilize it. It seems that there is an inverse association between the ability of spermatozoa to bind to HA and chromosomal abnormalities in the spermatozoa (Mokanszki *et al*., 2012). In a study, it was found that HA binding test increased the chance of selecting a spermatozoon with a low amount of DNA fragmentation possibly optimizing the chance of pregnancy. A commercial kit has been developed thereby increasing the availability to the method (Parmegiani *et al*., 2010) (Parmegiani *et al*., 2012). However, in a recent meta‐analysis it was not found that HA binding test increases fertilization rates after ICSI (Beck‐Fruchter *et al*., 2016) and further research in the area is thus needed for this test to have a relevance in the fertility clinics.

### DNA fragmentation and pregnancy loss

Research continuously seems to focus on the possible association between sperm DNA fragmentation and recurrent pregnancy loss (Coughlan *et al*., 2015; Leach *et al*., 2015; Bareh *et al*., 2016; Zidi‐Jrah *et al*., 2016). Furthermore, an increasing interest has supervened regarding the types of fragmentations present in the DNA (Wei *et al*., 2015) and how DNA fragmentation can be reduced in cryopreservation (Ghorbani *et al*., 2016; Kably‐Ambe *et al*., 2016; Simonenko *et al*., 2016). Recent papers have shown a possible correlation between an increased amount of DNA fragmentation and some of the natural antioxidants present in the seminal plasma like superoxide dismutase (Wdowiak *et al*., 2015) and glutathione peroxidase (Dorostghoal *et al*., 2017).

In spite of the effort in the last couples of decades, there is still a long way to go within the field of DNA fragmentation in spermatozoa. Substantial amounts of reviews and meta‐analysis have been published, many of them imploring further studies with a controlled, randomized study population and more sensitive assays (Lewis *et al*., 2013; Palermo *et al*., 2014; Zhao *et al*., 2014; Osman *et al*., 2015).

**Figure 1:**
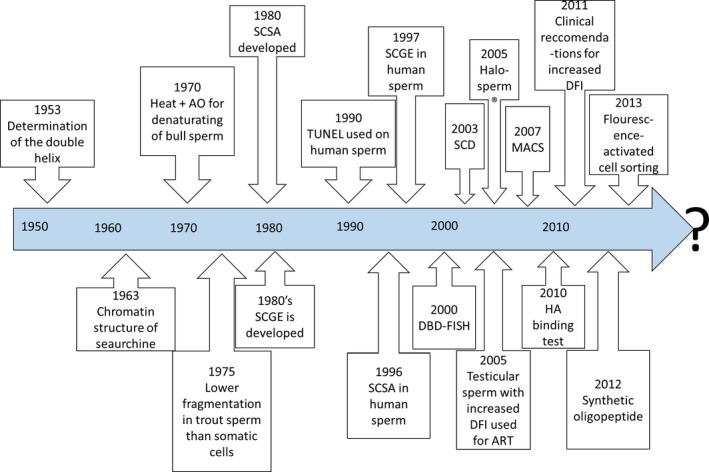
Timeline. An illustrative view of the landmarks and development.

In Fig. 1, an illustrative view of the historical development of DNA fragmentation in spermatozoa is presented.

## Discussion and Conclusion

In spite of half a century′s research and the widely accepted conviction that infertility and sperm DNA fragmentation are linked, this diagnostic tool is not yet a standard care in the fertility clinics.

Several issues contribute to this. The lack of uniformity in all assays, (except the SCSA) for analysis of DNA fragmentation and the absence of a clear clinical threshold, a myriad of studies using different assay, different clinical ART and diverse outcomes and small patient cohorts are all contributing factors. One of the obstacles is the difference in the methods used to assess the amount of DNA fragmentation present in the spermatozoa. Reviews and meta‐analysis compare outcomes of fertility treatment across methods, which impede the progress of implementing the analysis in the fertility clinics. Another obstacle is the lack in knowledge concerning the aetiology of DNA fragmentation. There are several theories of the aetiology of DNA fragmentation. One is a two‐step model for the development of DNA fragmentation in spermatozoa. In step one, an error in the spermatogenesis weakens the DNA and impairs the chromatin remodelling. This results in spermatozoa with low levels of nuclear protamine. In the second step, the vulnerable DNA is more susceptible to oxidative stress (Christensen & Birck, 2015). Another theory is that DNA fragmentation occurs after an interrupted apoptosis. An increased activity of apoptosis related proteins such as caspase 3 and 7, Fas and cPARP has been seen in samples with abnormal semen parameter or increased amount of DNA fragmentation. Apoptosis is believed to be activated by testicular conditions and oxidative stress. It is speculated that apoptosis could be the main pathway to DNA fragmentation in spermatozoa (Sakkas *et al*., 2004; Manente *et al*., 2015; Muratori *et al*., 2015). An association between chromatin immaturity and DNA fragmentation has also been seen; which can be suggested as an accelerator for DNA fragmentation. Chromatin immaturity is believed to be caused by defects in the spermatogenesis (Sati *et al*., 2008). The aetiology of DNA fragmentation is far from illuminated and the different theories are not mutually exclusive. It is possible that chromatin immaturity or oxidative stress triggers the activation of the apoptotic pathway. Furthermore, it is known that exogenous exposures, such as environment, lifestyle and health also contribute to increased amount of DNA fragmentation.

When relating to DNA fragmentation and infertility, the salient point is implementation in the fertility clinic. It is essential that this analysis is practicable in the daily work. Furthermore, uniformity and reproducibility across laboratories are of crucial importance. As the association between DNA fragmentation and pregnancy outcome is well established the analysis can be fully implemented as a diagnostic tool in the fertility clinics however, the aetiology of DNA fragmentation has not yet been fully elucidated.

When comparing outcomes of the different methods, there is a moderate correlation between SCSA, TUNEL and SCD with regard to levels of sperm DNA fragmentation. However, the Acridine Orange staining Technique (AOT) does not seem to have a clinical significance for fertility testing. In the AOT assay, the amount of DNA fragmentation is determined after a coloration with acridine orange and a microscopic evaluation. The method has recently been discredited by Evenson as a result of AO staining fluorescence fading and artefacts induced by glass/AO interactions (Evenson, 2016). Nonetheless SCSA also uses AO for the colouration of the DNA, the evaluation by microscopy vs. flow cytometer seems to be of crucial importance. Additionally, a study has shown that the neutral comet assay fails to distinguish between fertile donors and infertility patients. It does, however, relate to the risk of miscarriage. The alkaline comet assay seems to have a moderate correlation with SCSA, TUNEL and SCD. When the predictive values of the methods are assessed, there seems to be conflicting results regarding predictability for fertilization. It has been reported by Ribas‐Maynou in 2013 that the alkaline comet assay has the highest sensitivity followed by the TUNEL, SCD and SCSA analysis and subsequently the neutral comet assay. Chohan *et al*. found a strong relationship between SCSA and TUNEL (Chohan *et al*., 2006; Ribas‐Maynou *et al*., 2013). Furthermore, a systemic review and meta ‐analysis from 2016 claims that the comet assay and the TUNEL assay has the best predictability after IVF or ICSI of the methods assessed (Cissen *et al*., 2016).

Other tests, like SCD are available as an easy to use kit but might be less accurate. However, the comet assay has shown the best predictability in some studies, it lacks a clear threshold and the methodology can change among laboratories. Furthermore, the comet assay as well as the SCD test, suffers by the fact that the evaluation of DNA fragmentation is estimated in only 200–300 cells. In addition, the comet assay is labour‐intensive. The agreement between TUNEL and SCSA has been seen several times (Chohan *et al*., 2006; Ribas‐Maynou *et al*., 2013; Evenson, 2016). When comparing the two flow cytometric assays, the TUNEL assay requires extensive preparation of the spermatozoa before analysis can be performed and there is currently a lack of a strict protocol. This inhibits the implementation of this method as a diagnostic tool in a clinical setting. SCSA has a strict protocol developed in 1980 and has been used for fertility assessment in both animal and human spermatozoa. The protocol is relatively easy and the analysis is not time consuming. It is possible to analyse up to 50 samples per day for an experienced lab technician. The analysis by flow cytometer allows evaluation of 10.000 spermatozoa within a minute or two resulting in a more robust analysis (Evenson *et al*., 2002; Evenson & Wixon, 2006; Zini *et al*., 2009; Evenson, 2013).

In 2005, the SCSA test was commercialized. Two European laboratories received license to perform the SCSA test. Fertility clinics were to ship sperm samples to these larger diagnostic centres in order to obtain a SCSA‐DFI value (Evenson, 2011). As it is encouraged that DFI is determined using the commercial SCSAsoft^®^ software the SCSA analysis have primarily been restricted to the larger licensed diagnostic laboratories (Evenson, 2011). In order for the analysis to be implemented into the individual fertility clinics, an investment in a flow cytometer is required. This has previously been an insurmountable cost. Recently, smaller bench‐top flow cytometers having only a red and green fluorescent channel and a small air‐cooled blue laser has been developed. These are cheaper and require less space than the larger multichannel flow cytometers, making them more suitable for the smaller fertility clinics. More flexibility concerning software as well as a relocation from the larger commercial diagnostic labs to the individual fertility clinic will decrease the costs concerning the analysis thereby aiding the implementation of the analysis in the fertility clinics.

Bungum *et al*. (2011) estimates that 40% of all cases of unexplained infertility can be related to increased amount of DNA damage and suggests a treatment course where patients with DFI ≥30% measured by SCSA should be referred directly to IVF/ICSI treatment. Furthermore, it is speculated that even a moderate increase in DFI (between 20–30% by SCSA) can give rise to a prolonged TTP – information that the treating physician can employ when counselling fertility patients and planning the course of treatment (Bungum *et al*., 2011). As mentioned in the introduction, DNA fragmentation also seems to have implications for the offspring as it is linked to an increased risk of miscarriage or a number of pathogenic conditions in the offspring (reviewed in Aitken *et al*., 2009; Sills & Christensen, 2015). In order for progress within this field a necessary next step is a combination of further clarification of the aetiology of DNA fragmentation and simplifying and standardizing the analysis. While it is very important to standardize all sperm fragmentation assays for utility in the ART clinic, a better understanding on the aetiology of sperm DNA fragmentation will be needed in order to develop effective therapeutic strategies for these patients. For couples where the male suffers from increased amount of DNA fragmentation, ICSI or TESA are currently the only methods that have shown any positive effect on the pregnancy rates. Both procedures are invasive and cannot cope with the increased amount of early pregnancy loss also seen in this group of patients. The goal must be to ease the diagnosis of these patients and to clarify the origin of the DNA fragmentation. This will make it possible to plan a treatment course with the aim of reducing the amount of DNA fragmentation and increase the rate of continuous pregnancy for these couples.

The predictive value of the analysis of DNA fragmentation in spermatozoa is often criticized. One point of criticism is that the method cannot predict all failure to conceive. As infertility is the couple's problem, one has to consider the fertility of the female as well. One single test of gamete dysfunction from just one partner of the couple cannot predict the outcome of the fertility treatment. Determination of DNA fragmentation is not a replacement of current diagnostic tools for infertility diagnosing. However, it is a valuable supplement adding independent information about the gamete status of the male partner and it is due time that this analysis becomes a standard tool in the fertility clinics.

## Conflict of Interest

The authors declare no conflict of interest.

## Authors' Contributions

JF and ASR were responsible for idea, design and outline of content of the paper. ASR wrote the manuscript draft. All authors revised the manuscript critically and approved the final version.
